# Longitudinal associations between relative deprivation and non-suicidal self-injury in early adolescents: a moderated mediation model

**DOI:** 10.3389/fpsyt.2025.1553740

**Published:** 2025-03-21

**Authors:** Chang Wei, Bao Liu, Xiaojing An, Yu Wang

**Affiliations:** ^1^ School of Arts and Sciences, Guangzhou Maritime University, Guangzhou, China; ^2^ Student Affairs Department, North China Institute of Aerospace Engineering, Langfang, China; ^3^ Education Science Teaching Department, Xingtai University, Xingtai, China; ^4^ Research Center of Rural Educational and Cultural Development of the Key Research Institute of Humanities and Social Sciences in Hubei Province/School of Education, Hubei University of Science and Technology, Xianning, China

**Keywords:** early adolescents, relative deprivation, emotional symptoms, deviant peer affiliation, non-suicidal self-injury

## Abstract

**Introduction:**

Relative deprivation is associated with non-suicidal self-injury; however, the mechanisms underlying this association have been largely unexplored. Based on relative deprivation theory, the functional model of non-suicidal self-injury, and the organism-environment interaction model, the current study examined the mediating role of emotional symptoms and moderating role of deviant peer affiliation to determine how and when relative deprivation is associated with non-suicidal self-injury.

**Methods:**

Participants were 601 Chinese early adolescents who completed self-report scales measuring relative deprivation, emotional symptoms, non-suicidal self-injury, and deviant peer affiliation at three time points over the course of 12 months.

**Results:**

The results showed that relative deprivation at Wave 1 was positively associated with non-suicidal self-injury at Wave 3, and emotional symptoms at Wave 2 fully mediated this association. Furthermore, deviant peer affiliation at Wave 3 moderated the pathway from emotional symptoms to subsequent non-suicidal self-injury in the mediated model. Specifically, emotional symptoms significantly predicted an increase in non-suicidal self-injury but only in early adolescents with high deviant peer affiliation.

**Conclusions:**

Our findings can encourage educators to consider the interaction between individual and peer factors when providing interventions for early adolescents who engage in non-suicidal self-injury.

## Introduction

Non-suicidal self-injury refers to the direct and deliberate destruction of one’s own body tissue without suicidal intention (1). Previous research indicates that the global incidence of non-suicidal self-injury among adolescents is approximately 17.2% (2). In a recent study, Jiao (3) found that the prevalence of non-suicidal self-injury was 40.1% in a community sample of 4471 Chinese primary and secondary school students. Moreover, the onset of non-suicidal self-injury during childhood is an important predictor of future severe non-suicidal self-injury and suicidal behaviors (4, 5). Therefore, identifying the risk factors for non-suicidal self-injury in early adolescents may assist in developing targeted prevention and intervention efforts.

### Relative deprivation and non-suicidal self-injury

Relative deprivation refers to an individual’s subjective perception of being disadvantaged and experiencing negative emotions in comparison to a reference group (6, 7). Relative deprivation theory posits that individuals evaluate their situation and status by comparing themselves with others. Accordingly, feelings of relative deprivation can damage psychological development (8), which in turn leads to various emotional symptoms (e.g., depressive symptoms) (9) and deviant behaviors (e.g., non-suicidal self-injury) (10). Previous research supports the relationship between relative deprivation and non-suicidal self-injury (11, 12). For example, in a sample of 4861 Chinese adolescents, Hao et al. (11) found that relative deprivation was significantly positively associated with non-suicidal self-injury.

These studies have demonstrated a direct link between relative deprivation and non-suicidal self-injury. However, the underlying mechanism of this association re-mains largely unexplored. Therefore, this study examined whether the relationship between relative deprivation and non-suicidal self-injury is mediated by emotional symptoms and, if so, whether this mediating process is moderated by deviant peer affiliation.

### Emotional symptoms as a potential mediator

The first part of the mediation pathway tested in this study is the association between relative deprivation and emotional symptoms. According to the theory of relative deprivation, individuals perceive themselves to be at a disadvantage through comparison with others, which in turn generates a variety of emotional symptoms such as anxiety and depression. According to the relative deprivation theory, individuals perceive themselves to be disadvantaged by comparing themselves to others, which in turn leads to various emotional symptoms such as anxiety and depression (8, 9, 12). Empirical evidence indicates that relative deprivation may increase the risk of emotional symptoms in children and adolescents (9, 13). For example, in a longitudinal study of 273 Chinese children and adolescents, Xiong et al. (9) found that relative deprivation was positively correlated with depressive symptoms at 6- and 12-month follow-up.

The second part of the mediation pathway is the association between emotional symptoms and non-suicidal self-injury.

According to the functional model of non-suicidal self-injury, non-suicidal self-injury is a maladaptive means of emotion regulation that can immediately alleviate or stop an individual’s emotional symptoms (1, 14). Several studies have shown a link between emotional symptoms and non-suicidal self-injury (15–17). In a longitudinal study, Gao et al. (16) found that depressive symptoms were correlated with non-suicidal self-injury at six-month follow-up in Chinese rural-to-urban children (Mage = 11.95).

Furthermore, multiple studies have demonstrated the mediating role of emotional symptoms in the association between relative deprivation and problem behaviors (18, 19). For example, Wu (19) found that negative emotions mediated the relationship between relative deprivation and aggressive behavior in a sample of 500 college students in China. Thus, based on theory and empirical research, we propose the following hypothesis:

Hypothesis 1: Emotional symptoms mediate the association between relative deprivation and non-suicidal self-injury in early adolescents.

### Deviant peer affiliation as a moderator

Although relative deprivation may be related to non-suicidal self-injury through emotional symptoms, it may not affect all early adolescents equally. Therefore, the potential moderators of this relationship must be explored. Previous research has shown that peers play an important role in shaping the behavior of children and adolescents (20, 21). Deviant peer affiliation refers to selective affiliation with peers who engage in deviant behaviors, such as tobacco and alcohol use, truancy, and delinquency (22, 23). According to the organism-environment interaction model (24), the development of individual behavior (e.g., non-suicidal self-injury) is determined by the interaction of individual (e.g., relative deprivation, emotional symptoms) and environmental (e.g., deviant peer affiliation) factors.

Consistent with this view, Tian et al. (25) found that deviant peer affiliation interacted with self-control ability to predict risk-taking behavior in a sample of 1263 adolescents. Specifically, the effect of low self-control ability on risk-taking behavior was stronger for adolescents with higher deviant peer affiliation than for those with lower deviant peer affiliation. Similarly, Wang et al. (26) found that deviant peer affiliation interacted with self-esteem to predict negative risk-taking behavior in a sample of 940 adolescents. Empirical research has demonstrated that deviant peer affiliation is an important risk factor for non-suicidal self-injury (21). Therefore, based on theory and empirical research, we propose the following hypothesis:

Hypothesis 2: Deviant peer affiliation strengthens the direct and indirect effects of relative deprivation on non-suicidal self-injury in early adolescents.

### The present study

**Figure 1:**
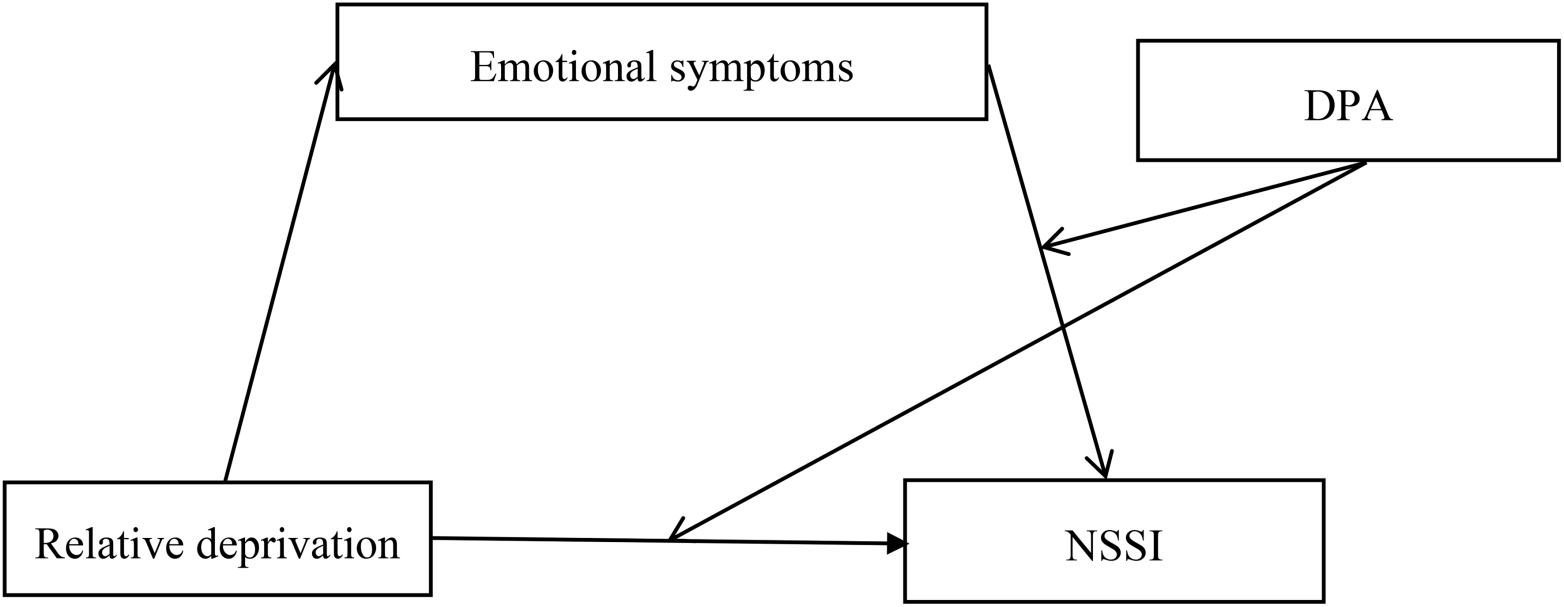
The proposed moderated mediation model note. DPA, deviant peer affiliation; NSSI, non-suicidal self-injury.

Based on relative deprivation theory (8), the function model of non-suicidal self-injury (1, 14) and the organism-environment interaction model (24), we considered emotional symptoms and deviant peer affiliation as a mediator and moderator, respectively, to explain how and when relative deprivation is associated with early adolescents’ non-suicidal self-injury. Specifically, we hypothesized that emotional symptoms mediate the link between relative deprivation and non-suicidal self-injury (Hypothesis 1) and that deviant peer affiliation moderates the direct and indirect relationships between relative deprivation and non-suicidal self-injury (Hypothesis 2). **Figure 1** presents the proposed model.

## Method

### Participants

We recruited the participants from two primary schools in Hubei Province, China. A total of 631 early adolescents participated in the first wave of the questionnaire. Due to reasons such as school transfers and absences, 30 participants were lost during the second and third waves. Ultimately, 601 early adolescents were identified as the subjects of this study. T-tests revealed no significant differences between the attrition group and the final sample on any of the main variables (*Ps* > 0.05). Of the participants, 50.2% were boys and 49.8% were girls. Additionally, 46.9% of the participants’ fathers and 48.9% of their mothers had less than a high school or secondary vocational and technical school education, and 55.9% of the participants were from rural areas.

### Procedure

Inclusion Criteria: Written informed consent provided by parents and consent to participation by adolescents. Exclusion Criteria: Students who do not agree to participate in the questionnaire or who are not in school due to leave are excluded. They were told that they could withdraw from the study at any time without penalty and that their responses would be kept confidential. All participants were given a signature pen as a token of appreciation.

### Measures

#### Relative deprivation

At Wave 1, relative deprivation was measured using the Relative Deprivation Scale (27). This scale includes 10 items (such as “Compared to my classmates, their family has better financial conditions”) rated on a 5-point scale (1 = almost always untrue to 5 = almost always true). The responses were averaged across the 10 items, with higher scores indicating higher relative deprivation. In this study, Cronbach’s alpha was.88 at Wave 1.

#### Emotional symptoms

At both Wave 1 and Wave 2, emotional symptoms were measured using the Emotional Symptoms Subscale of the Chinese Version of the Strengths and Difficulties Questionnaire (28), which was adapted from the Strengths and Difficulties Questionnaire (29). This scale includes five items (such as “I was often unhappy, heavy or in tears”) rated on a 3-point scale (0 = untrue to 2 = always true). The responses were averaged across the five items, with higher scores indicating higher levels of emotional symptoms. In this study, Cronbach’s alpha was.83 and.87 at Waves 1 and 2, respectively.

#### Deviant peer affiliation

At Wave 3, deviant peer affiliation was measured using the Deviant Peer Affiliation Scale (30). This scale includes 10 items (such as “online addiction”) rated on a 5-point scale (1 = none to 5 = six or more). The responses were averaged across the 10 items, with higher scores indicating higher deviant peer affiliation. In this study, Cronbach’s alpha was.77 at Wave 3.

#### Non-suicidal self-injury

At both Wave 1 and Wave 3, non-suicidal self-injury was measured with assessed twelve non-suicidal self-injury behaviors (31) selected from the Deliberate Self-Harm Inventory Scale (32). This scale includes 12 items (such as “cutting yourself”) rated on a 6-point scale (0 = never to 5 = 5 or more times). The responses were averaged across the 12 items, with higher scores indicating higher non-suicidal self-injury. In this study, Cronbach’s alpha was.85 and.89 at Waves 1 and 3, respectively.

### Statistical analyses

We used SPSS 21.0 to generate descriptive statistics and correlations. We adopted Model 4 of the PROCESS for SPSS proposed by Hayes to examine whether emotional symptoms at Wave 2 mediated the association between relative deprivation at Wave 1 and non-suicidal self-injury at Wave 3. To further test the moderating effect of deviant peer affiliation at Wave 3 on the direct and indirect relationships between relative deprivation at Wave 1 and non-suicidal self-injury at Wave 3, we used Model 15 of PROCESS for SPSS for data processing. Previous studies have shown that gender is significantly associated with non-suicidal self-injury, with female adolescents at a higher risk (33). Additionally, non-suicidal self-injury is also associated with age (2). Therefore, the present study incorporated gender and age as covariates in the analysis.

## Results

### Preliminary analyses

**Table 1** shows the means, standard deviations, and correlation coefficients for all study variables. Relative deprivation at Wave 1 was positively associated with non-suicidal self-injury at Wave 3 and emotional symptoms at Wave 2. In addition, emotional symptoms at Wave 2 were positively associated with non-suicidal self-injury at Wave 3.

**Table 1:** Descriptive statistics and correlations for all variables.

Variable	1	2	3	4	5	6	7	8	9
1. Gender	1.00								
2. Age	0.12***	1.00							
3. RD	-0.02	0.06	1.00						
4.ES at Wave 1	-0.07	0.02	0.41***	1.00					
5. ES at Wave 2	-0.15***	0.01	0.29***	0.42***	1.00				
6. NSSI at Wave 1	0.01	0.13**	0.17***	0.32***	0.21***	1.00			
7. NSSI at Wave 2	-0.05	0.04	0.16***	0.18***	0.26***	0.42***	1.00		
8. NSSI at Wave 3	-0.02	0.03	0.10*	0.14***	0.23***	0.34***	0.71***	1.00	
9. DPA at Wave 3	0.03	0.00	0.16***	0.14***	0.20***	0.15***	0.22***	0.22***	1.00
*Mean*	0.50	10.21	2.10	0.42	0.48	0.13	0.11	1.10	1.18
*SD*	0.50	0.73	0.83	0.55	0.57	0.36	0.36	0.38	0.32

Gender was dummy coded as 1 = male, 0 = female. RD, relative deprivation; ES, emotional symptoms; DPA, deviant peer affiliation; NSSI, non-suicidal self-injury. *p <.05. **p <.01. ***p <.001.

### Mediation effect of emotional symptoms

**Figure 2:**
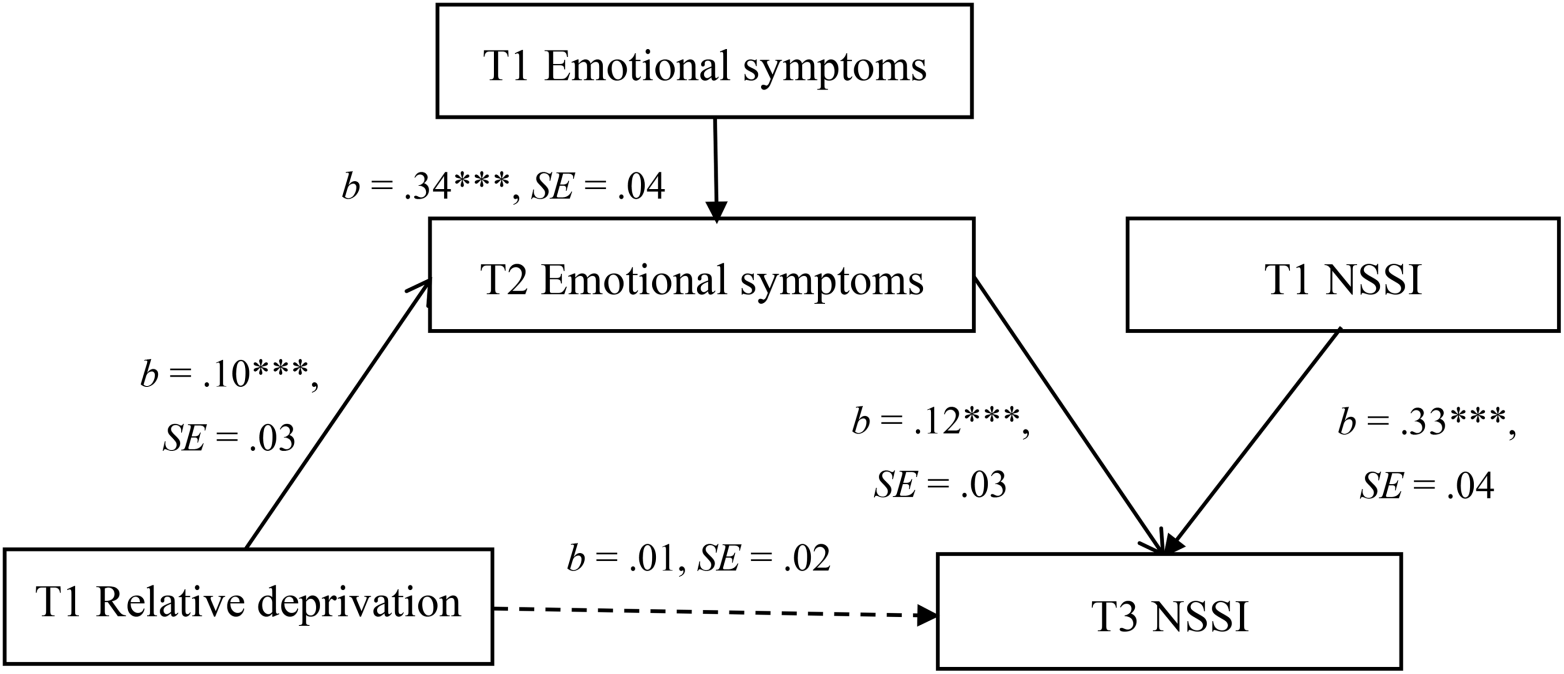
Model of the mediating role of emotional symptoms in the association between relative deprivation and non-suicidal self-injury. The mediational analysis of emotional symptoms at Wave 2 in the association between relative deprivation at Wave 1 and non-suicidal self-injury at Wave 3. The model also includes age and gender as controlled variables which are not displayed. NSSI, non-suicidal self-injury. The dashed line indicates that the relationship is not significant. ***p <.001. .

**Figure 2** shows the results obtained from the mediation model. After controlling for gender, age, emotional symptoms, and non-suicidal self-injury at Wave 1, relative deprivation at Wave 1 positively predicted emotional symptoms at Wave 2 (*b* = .10, *SE* = .03, *p* <.001, 95% CI = [0.044, 0.154]), which in turn positively predicted non-suicidal self-injury at Wave 3 (*b* = .12, *SE* = .03, *p* <.001, 95% CI = [0.065, 0.180]). The bias-corrected percentile bootstrap method showed a significant mediating effect of emotional symptoms at Wave 2 in the relationship between relative deprivation at Wave 1 and non-suicidal self-injury at Wave 3 (indirect effect = .012, *SE* = .005, 95% CI = [0.003, 0.024]).

In addition, the sex differences in the model were further examined, and the mediation model was only significant in the female sample. The results showed that relative deprivation at Wave 1 positively predicted emotional symptoms at Wave 2 (*b* = .11, *SE* = .04, *p* <.05, 95% CI = [0.026, 0.198]), which in turn positively predicted non-suicidal self-injury at Wave 3 (*b* = .14, *SE* = .04, *p* <.001, 95% CI = [0.069, 0.212]). The bias-corrected percentile bootstrap method showed a significant mediating effect of emotional symptoms at Wave 2 in the relationship between relative deprivation at Wave 1 and non-suicidal self-injury at Wave 3 (indirect effect = .016, *SE* = .010, 95% CI = [0.002, 0.041]).

### Moderated mediation

**Figure 3:**
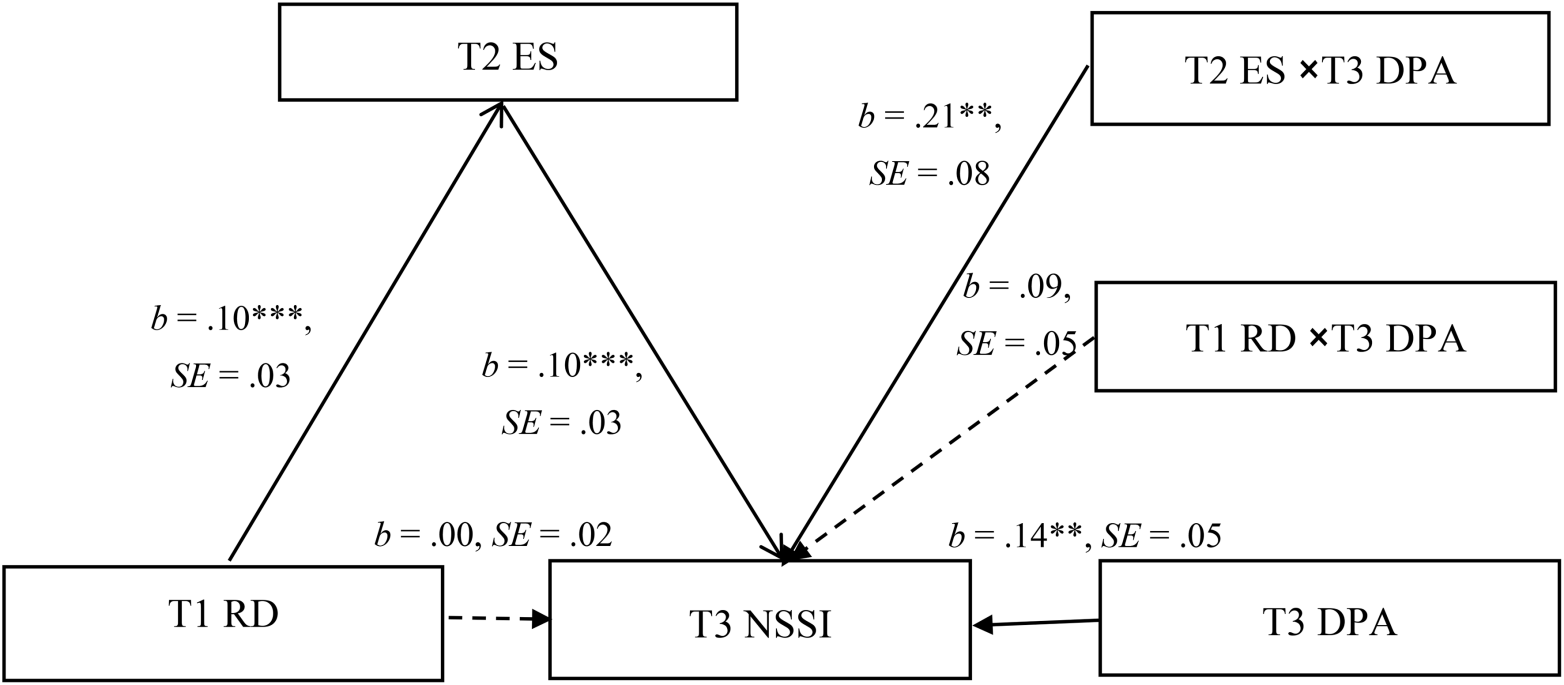
Model of the moderating role of deviant peer affiliation on the direct and indirect relationship between relative deprivation and non-suicidal self-injury. The model also includes age and gender as controlled variables which are not displayed. RD, relative deprivation; ES, emotional symptoms; DPA, deviant peer affiliation; NSSI, non-suicidal self-injury. The dashed line indicates that the relationship is not significant. **p <.01. ***p <.001. .

**Figure 4:**
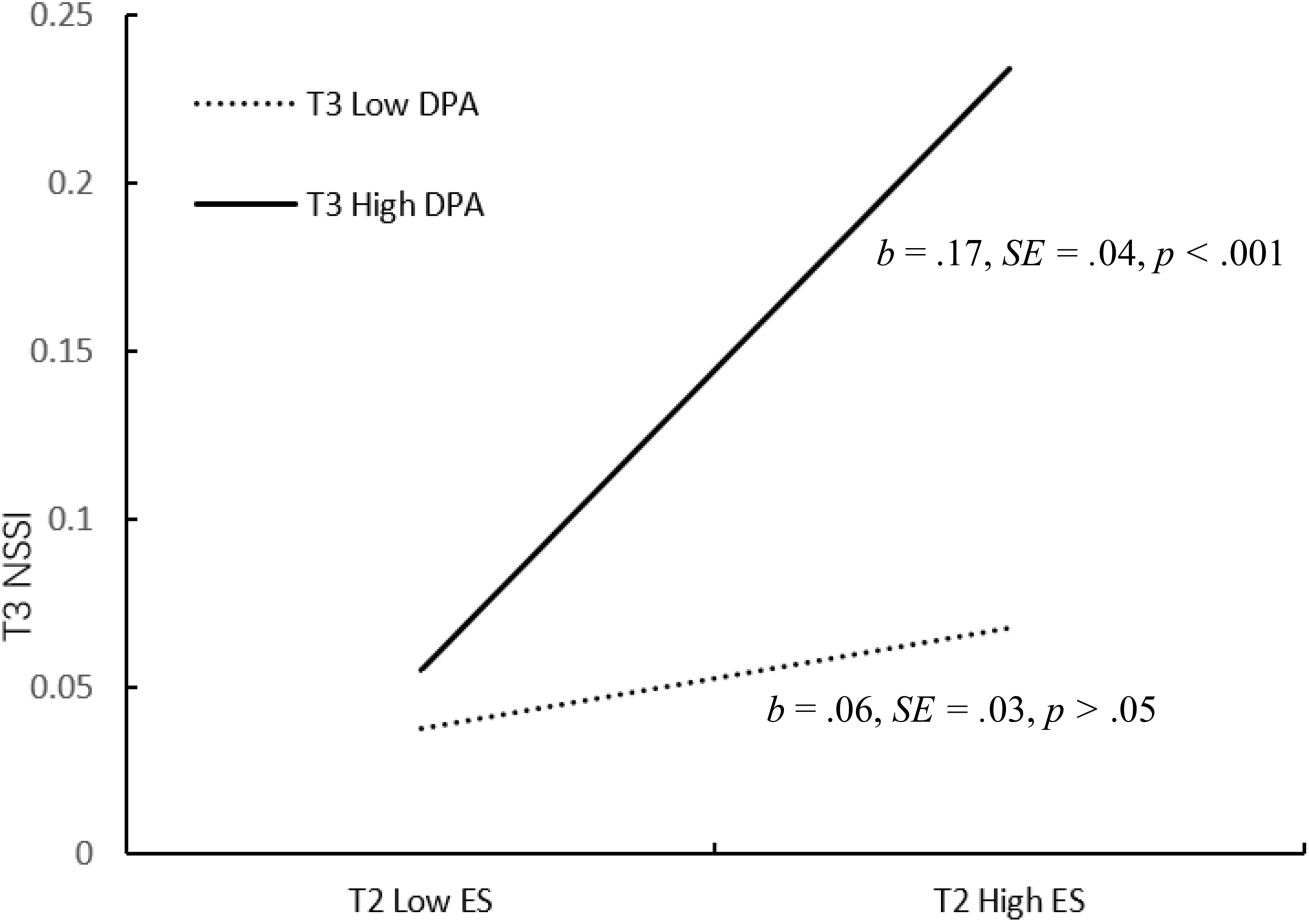
Interactive effect of emotional symptoms and deviant peer affiliation on non-suicidal self-injury. Deviant peer affiliation at Wave 3 as a moderator of the relationship between emotional symptoms at Wave 2 and non-suicidal self-injury at Wave 3. ES, emotional symptoms; DPA, deviant peer affiliation; NSSI, non-suicidal self-injury.

**Figure 3** shows the results of the moderated mediation model. After controlling for gender, age, emotional symptoms, and non-suicidal self-injury at Wave 1, the results showed that deviant peer affiliation at Wave 3 moderated the association between emotional symptoms at Wave 2 and non-suicidal self-injury at Wave 3 (*b* =.21, *SE* = .08, *p* <.01, 95% CI = [0.052, 0.364]). We conducted simple slope tests to better understand the results using deviant peer affiliation as a moderator. As shown in **Figure 4**, when participants reported high deviant peer affiliation at Wave 3, the association between emotional symptoms at Wave 2 and non-suicidal self-injury at Wave 3 was significant (*b* = .17, *SE* = .04, *p* <.001, 95% CI = [0.097, 0.242]). However, for participants who reported low deviant peer affiliation at Wave 3, this association was not significant (*b* = .06, *SE* = .03, *p* >.05, 95% CI = [-0.005, 0.129]). Therefore, the mediating effect of emotional symptoms on the association between relative deprivation and non-suicidal self-injury was significant in early adolescents with high deviant peer affiliation.

In addition, the sex differences in the model were further examined, and the moderated mediated model was only significant in the female sample. The results showed that deviant peer affiliation at Wave 3 moderated the association between emotional symptoms at Wave 2 and non-suicidal self-injury at Wave 3 (*b* =.45, *SE* = .11, *p* <.001, 95% CI = [0.222, 0.669]). We conducted simple slope tests to better understand the results using deviant peer affiliation as a moderator. When participants reported high deviant peer affiliation at Wave 3, the association between emotional symptoms at Wave 2 and non-suicidal self-injury at Wave 3 was significant (*b* = .26, *SE* = .05, *p* <.001, 95% CI = [0.165, 0.361]). However, for participants who reported low deviant peer affiliation at Wave 3, this association was not significant (*b* = .05, *SE* = .04, *p* >.05, 95% CI = [-0.035, 0.128]).

## Discussion

Although previous research has demonstrated the association between relative deprivation and non-suicidal self-injury (11, 12)), the reasons for this association have been unclear. Based on relative deprivation theory (8), the function model of non-suicidal self-injury (1, 14), and the organism–environment interaction model (24), we examined the mediating and moderating roles of emotional symptoms and deviant peer affiliation, respectively, to clarify how and when relative deprivation is associated with non-suicidal self-injury in early adolescents. As expected, we found that emotional symptoms mediated the association between relative deprivation and non-suicidal self-injury and that the indirect effect was strengthened by deviant peer affiliation.

### The mediating role of emotional symptoms

The findings supported Hypothesis 1, which posits that emotional symptoms mediate the association between relative deprivation and non-suicidal self-injury. Specifically, emotional symptoms fully mediated the direct association between relative deprivation and non-suicidal self-injury. This shows that emotional symptoms are a key bridge linking relative deprivation to non-suicidal self-injury in early adolescents. One possible explanation is that relative deprivation may weaken an individual’s social support network, which in turn affects emotional symptoms (34). Furthermore, emotional symptoms are an important trigger for non-suicidal self-injury in adolescents (16). Therefore, relative deprivation may lead to early adolescents’ emotional symptoms, which in turn could increase their risk of non-suicidal self-injury. These findings align with relative deprivation theory (8), which argues that relative deprivation can affect individual’s psychological development, leading to the onset of emotional symptoms. Moreover, our results are consistent with those of a previous study showing that early adolescents experience emotional symptoms when they feel disadvantaged (9).

The function model of non-suicidal self-injury (1, 14) suggests that it is a maladaptive coping strategy that individuals use to alleviate emotional symptoms. Previous studies have found that emotional symptoms are an important risk for non-suicidal self-injury in children and adolescents (15, 16). In the current study, early adolescents who reported higher levels of relative deprivation were more likely to develop emotional symptoms after six months, which in turn led to non-suicidal self-injury six months later. Our study used a longitudinal design to demonstrate the indirect role of emotional symptoms in the association between relative deprivation and non-suicidal self-injury. Thus, our findings extend those of previous studies on the mediating role of emotional symptoms in the association between relative deprivation and problem behaviors (18, 19). In summary, this study, through its longitudinal design, has revealed the mediating role of emotional symptoms between relative deprivation and non-suicidal self-injury, offering new insights into understanding non-suicidal self-injury in adolescents.

### The moderating role of deviant peer affiliation

Consistent with Hypothesis 2, deviant peer affiliation strengthened the relation-ship between emotional symptoms and non-suicidal self-injury (the second part of the indirect pathway), and further increased the indirect association between relative deprivation and subsequent non-suicidal self-injury. Specifically, emotional symptoms significantly predicted an increase in non-suicidal self-injury but only in early adolescents with higher deviant peer affiliation. A possible explanation for this is that early adolescents with higher deviant peer affiliation are more likely to use rash maladaptive adjustment strategies, such as non-suicidal self-injury (21), to alleviate emotional symptoms triggered by relative deprivation.

According to the social learning hypothesis (35), adolescents may learn maladaptive behaviors, such as non-suicidal self-injury, by observing their peers (1). Prior research has confirmed that deviant peer affiliation is associated with a higher likelihood of non-suicidal self-injury among adolescents (36). Our findings are also congruent with the organism–environment interaction model (24) and provide further evidence that higher deviant peer affiliation can strengthen the indirect mechanism through which relative deprivation leads to non-suicidal self-injury in early adolescents. These results align with those of previous studies that found that deviant peer affiliation is an important peer environmental risk factor (37, 38) that can strengthen the association between personality vulnerability (e.g., low self-control and low self-esteem) and adolescent risk behaviors (25, 26). Furthermore, our findings also support the view that peers play a key role in adolescents’ behavioral development (20).

### Limitations and future directions

This study had several limitations. First, the data in this study came from early adolescents’ self-reports, which provide subjective information. Future research should collect data from multiple sources (e.g., parents or teachers), to provide a more comprehensive understanding of the behaviors involved. Second, the sample for this study was drawn from early adolescents in the central region of China, which limits the generalizability of the results due to the demographic and cultural characteristics of the sample. Future studies should recruit samples from different regions and cultural to test the generalizability of the findings. Third, the type of emotional symptoms (e.g., anxiety and depression) may have affected our results. Future studies should focus on different types of emotional symptoms to further validate our moderated mediation model. Fourth, this study employed a quantitative research method, which, while providing quantifiable data on adolescent non-suicidal self-injury and its related factors, limits our in-depth understanding of the underlying context and motivations. Future research could benefit from incorporating qualitative methods, such as interviews and open-ended questions, to enhance the depth and contextual understanding of the study.

### Implications for practice

Implications for practice. This study has several important implications for prevention and intervention strategies. First, given that emotional symptoms are a mechanism that links relative deprivation to non-suicidal self-injury, reducing emotional symptoms may help prevent the negative influence of relative deprivation on non-suicidal self-injury in early adolescents. Previous research has suggested that teaching interpersonal communication skills and emotional regulation methods is effective in alleviating adolescents’ emotional symptoms (39, 40). Second, we found a moderating effect of deviant peer affiliation on the pathway from emotional symptoms to subsequent non-suicidal self-injury, suggesting that reducing deviant peer affiliation may attenuate the risk of relative deprivation on non-suicidal self-injury. Prior research suggests that deviant peer affiliation can be reduced by improving adolescents’ self-control (41). For example, teaching emotion management methods can be used to enhance adolescents’ self-control abilities (42), thereby enabling them to resist the influence of deviant peers and reducing the risk of non-suicidal self-injury.

## Conclusions

The current study provides longitudinal evidence of how relative deprivation affects subsequent non-suicidal self-injury in Chinese. The results showed that emotional symptoms were a fully explanatory mechanism by which relative deprivation was associated with non-suicidal self-injury over time. In addition, deviant peer affiliation moderated this mediation process. Specifically, compared to early adolescents with lower deviant peer affiliation, those with higher deviant peer affiliation were more likely to engage in non-suicidal self-injury when experiencing high emotional symptoms. Thus, higher deviant peer affiliation is a key risk factor that can amplify the impact of high emotional symptoms on non-suicidal self-injury in early adolescents.

## Data Availability

The original contributions presented in the study are included in the article/supplementary material. Further inquiries can be directed to the corresponding authors.
